# A cross-sectional survey to explore healthcare providers’ experiences and attitudes toward HIV pre-exposure prophylaxis for women in family planning centers of Greater Paris

**DOI:** 10.1371/journal.pone.0337510

**Published:** 2026-01-16

**Authors:** Geoffroy Liegeon, Joseph A. Mason, Eleanor E. Friedman, Myriam Toribio, Sophie Florence, Elena Villalon, Julie Castaneda, Andrés Ramírez Zamudio, Samantha A. Devlin, Jessica P. Ridgway, Amy K. Johnson, Victoria Manda

**Affiliations:** 1 Section of Infectious Diseases and Global Health, Department of Medicine, University of Chicago, Chicago, Illinois, United States of America; 2 Department of Infectious Diseases, Assistance Publique – Hôpitaux de Paris, Saint-Louis and Lariboisière Hospitals, Paris, France; 3 Section of Maternal Protection, Family Planning, and Sexual Health Promotion, Department of Seine-Saint-Denis, Seine-Saint-Denis, France; 4 Department of Sexual Health, Public Health Service of Paris, Paris, France; 5 Berthe-Morisot Family Planning Center, Montfermeil, France; 6 Department of Gynecology and Obstetrics, Assistance Publique – Hôpitaux de Paris, Lariboisière Hospital, Paris, France; 7 The Potocsnak Family Division of Adolescent and Young Adult Medicine, Ann and Robert H. Lurie Children’s Hospital, Chicago, Illinois, United States of America; 8 Feinberg School of Medicine, Northwestern University, Chicago, Illinois, United States of America; Indiana University Bloomington, UNITED STATES OF AMERICA

## Abstract

Despite representing a disproportionately high percentage of new HIV diagnoses in France annually, women who have migrated from Sub-Saharan Africa (WMSSA) remain underserved by HIV prevention strategies, including Pre-Exposure Prophylaxis (PrEP). This study aimed to understand healthcare providers’ experiences and attitudes toward PrEP delivery to WMSSA within family planning centers (FPCs) of the Paris region in France. We conducted a web-based cross-sectional survey from February to June 2024 to explore the knowledge, attitudes, and experiences of providers in FPCs in Paris and Seine-Saint-Denis (SSD) County. The survey link was emailed to FPC providers via their departmental mailing lists. Of the 284 providers who were contacted, 64 completed the survey (response rate of 23%). Respondents were predominantly women (95%), with a median age of 44 (IQR 35–53) and a median of 17.5 (IQR 10–26) years of professional experience. They worked as physicians (44%), midwives (34%), or nurses (22%), primarily in FPCs within SSD County (77%). All providers had heard of PrEP; 42% had already discussed it with a client; 28% reported PrEP prescriptions being offered in their FPC; and 21% had already prescribed it for a woman. Among participants, 42% had received PrEP training, and 53% rated their overall PrEP knowledge as good or very good. About one-third of providers reported feeling uncomfortable discussing or prescribing PrEP to women. The top three barriers to PrEP implementation were the lack of PrEP awareness among clients (32%), inadequate provider training (21%), and the limited number of PrEP prescribers in FPCs (21%). Providers endorsed multiple interventions to increase PrEP delivery, including PrEP training, educational materials, and policy shifts to broaden prescriber roles. FPC providers in Paris and SSD County have limited experience in delivering PrEP to women. Several facilitators were identified to inform PrEP implementation strategies at the provider, client and structural levels.

## Introduction

In 2023, one in three new diagnoses of HIV in France occurred among cisgender women (hereafter referred to as “women”) [1]. Despite overall progress in HIV prevention, HIV incidence among women has remained largely unchanged for the past decade, contrasting with a marked decline among men who have sex with men (MSM) born in France [1]. This disparity is especially pronounced among women who have migrated from Sub-Saharan Africa (WMSSA), who represent nearly 80% of new HIV infections among women in France [1]. HIV acquisition risk is heightened during the post-migration settlement period, when women often face precarious living conditions, lack of legal residence, unstable housing, and limited economic resources—factors associated with increased exposure to sexual violence, transactional relationships and inconsistent condom use [2–5]. As a result, an estimated 31–62% of HIV infections are acquired post-arrival in Europe [2,6–8]. These structural and social hardships render WMSSA particularly vulnerable to HIV acquisition, underscoring the urgent need for targeted prevention strategies.

Pre-exposure prophylaxis (PrEP) is a highly effective biomedical intervention for preventing HIV transmission [9–11]. Oral PrEP with tenofovir disoproxil fumarate and emtricitabine (TDF/FTC) has been approved by the European Medicines Agency since 2016. Despite it being available and fully subsidized in France since that date, major PrEP access disparities persist, with women accounting for only 3% of the 103,000 people who initiated PrEP between January 2016 and June 2024 in the country [12]. This pattern is also observed in other European countries like the United Kingdom, where Black/African women remain the most underserved community in terms of PrEP use, demonstrating the lowest PrEP-to-need ratio (number of PrEP users divided by new HIV diagnoses) [13].

As the current PrEP delivery models have failed to effectively reach women, alternative approaches are needed to fill the PrEP access and delivery gap in this population. One potential model is the integration of PrEP into reproductive health services, which has been effective in increasing PrEP uptake in sub-Saharan Africa [14–16]. However, this strategy also presents significant challenges. At the individual level, potential barriers include lack of knowledge or training among providers, low awareness of PrEP among women, and inadequate time for PrEP screening, counseling and provision during appointments. At the structural level, the lack of dedicated space, resources, or infrastructure to support providers, and the regulatory or policy obstacles have also been shown to be potential barriers [16–19].

To date, there has been limited research in France and other European countries regarding the integration of PrEP delivery into family planning centers (FPCs) as a strategy to enhance PrEP access and uptake among WMSSA. Little is known about existing knowledge and practices, the feasibility and acceptability of PrEP delivery in FPCs, and the barriers and facilitators for implementation and scale-up. To address this gap, we conducted a survey to understand the knowledge, practices, barriers, and facilitators related to PrEP delivery among physicians, midwives, and nurses working in FPCs in two HIV transmission hotspots in Greater Paris, France.

## Materials and methods

### Study setting

The Île-de-France region (Paris and its suburbs) accounts for 40% of new HIV infections in France and is home to 56% of the country’s immigrants from sub-Saharan Africa [1]. We conducted this study in the network of public FPCs providing sexual and reproductive health services for women in the city of Paris and Seine-Saint-Denis (SSD) County, the two areas in the Île-de-France region with the highest HIV incidence and a large population of sub-Saharan African migrants. These FPCs are overseen by local public health departments, can be hospital-based or stand-alone, are open on weekdays, and may also serve populations other than women. All FPCs are primarily operated by midwives and nurses, with part-time support from physicians.

### Study design

We conducted a quantitative, web-based, self-administered, cross-sectional survey to evaluate knowledge, practices, barriers, and facilitators related to PrEP delivery among providers working in FPCs in Paris and SSD County. The survey was designed as part of the *PrEParez-vous!* study, a mixed-methods research project aimed at informing the development of an implementation strategy to increase PrEP uptake among WMSSA at the Paris and SSD County FPCs. The questionnaire included 68 questions across six sections: providers and clinical practice characteristics, PrEP awareness, PrEP knowledge, attitudes toward PrEP, barriers to PrEP use, and facilitators of PrEP use. As no validated questionnaires existed to explore this topic among providers, the survey was designed by the research team based on previous studies [20–23]. Self-rated knowledge about PrEP was measured on a five-point Likert scale (poor = 1, fair = 2, good = 3, very good = 4, excellent = 5). For items related to attitudes, perceived barriers, and facilitators, we used a 4-point Likert scale (not at all likely = 1, somewhat likely = 2, moderately likely = 3, extremely likely = 4) to avoid intermediate options and to reduce social desirability bias [24,25]. After drafting the questionnaire, we conducted a pilot test with four members of FPCs in Paris and SSD County, including two charge nurses, an experienced midwife, and a physician. The research team reviewed and incorporated the pilot test feedback to refine the questionnaire. The survey was conducted exclusively in French and took approximately 15 minutes to complete. An English translation is provided (S1 Survey). Providers were eligible for the survey if they met the following eligibility criteria: (i) 18 years of age or older, (ii) being a physician, midwife, or nurse, and (iii) working in a sexual and reproductive health center dedicated to women in Paris or SSD County.

### Data collection

We recruited potential participants through a dedicated mailing list from the Paris and SSD County public health departments. Recruitment emails, which included a brief description of the study and a link to the online survey, were sent by the heads of the Paris and SSD County public health departments. The mailing list contained 38 providers in Paris and 246 in SSD County. For participants who did not initially respond, reminder emails were sent monthly for 5 months, until the close of the survey. All participants confirmed their eligibility and signed an informed consent form before accessing the questionnaire. Data were collected between February and June 2024 using REDCap, a secure, web-based application designed for data capture in research studies. Participants accessed the survey on their personal computers, and REDCap automatically generated unique study identification numbers to ensure anonymity. All collected data were managed, stored, and processed in accordance with the European General Data Protection Regulation (GDPR) [26]. Upon completion of data collection, the dataset was exported from REDCap, cleaned, and subsequently analyzed by the study’s data analyst.

### Statistical analysis

Descriptive analyses on survey distribution, provider participation, provider characteristics, and study variables were conducted reporting frequencies and percentages (number, %) for categorical variables and medians and interquartile ranges (median, IQR) for continuous variables. In calculating survey response rate, only fully completed surveys were included. Survey response rates were compared between Paris and SSD County using a two-sample proportion Z-test with a sample size of 284 and statistical significance set at *p* ≤ 0.05. We also compared responses between physicians, midwives, and nurses on the following questions: “How effective do you think PrEP is in preventing the acquisition of HIV among women who take it every day as prescribed?” and “How would you rate your general knowledge of PrEP?,” using Chi-square test and Fisher’s exact tests (Fisher’s exact tests were used when cell sizes were less than 5). The Likert scale items were presented in absolute numbers and frequencies; scales were not developed to provide scores. Differences between professions were considered using a threshold *p *≤ 0.05 for statistical significance. The Consensus-Based Checklist for Reporting of Survey Studies (CROSS) was used as a guideline to report our results [27]. All data analysis was performed using R (version 4.0.3; R Core Team) [28].

### Ethics

This study was approved by the French ethics committee (Comité de Protection des Personnes Sud-Est 3, Date: 10/05/2023, No: 2023-A01817-38) and the Institutional Review Board at Ann & Robert H. Lurie Children’s Hospital of Chicago (Date: 10/27/2023, No: IRB 2024-6494). It also complied with the reference methodology MR-003 of the French National Commission on Informatics and Liberty (CNIL) (Deliberation no. 2018-153). The survey was anonymous and not incentivized.

### Inclusivity in global research

Additional information regarding the ethical, cultural, and scientific considerations specific to inclusivity in global research is included (S2 File).

## Results

### Response rate and participants characteristics

Of the 284 providers contacted, 97 opened the survey; 84 filled out the eligibility criteria; 83 were found eligible; 80 agreed to participate in the study; 67 initiated the questionnaire; and 64 fully completed it. Thus, the overall completion rate was 23% and was significantly higher in Paris (39%) than in Seine-Saint-Denis (20%) (*z* = 2.69, *p = *0.007). Participants included 28 physicians, 22 midwives, and 14 nurses. The median age (IQR) of participants was 44 (35–53) years, with the majority being cisgender women (95%). The median years of practice and years working in a FPC were 17.5 (IQR: 10–25.5) and 9 (IQR: 2–12) years, respectively. In terms of employment status, 41% worked full-time and 59% worked part-time. The median number of women seen per week by each provider in the FPCs was 25 (IQR: 15–40), regardless of provider type. Participant characteristics by job role are presented in Table 1.

**Table 1:** Providers’ characteristics.

Characteristics	Median (IQR), Number (%)			
	All providers (*N* = 64)	Physicians (*N* = 28)	Midwives (*N* = 22)	Nurses (*N* = 14)
**Age**	44 (35-53)	33 (35-54)	43 (38-49)	44.5 (36-52)
**Gender**				
Cisgender men	3 (5%)	3 (11%)	0	0
Cisgender women	61 (95%)	25 (89%)	22 (100)	14 (100)
**Years of practice**	18 (10-26)	15 (6-22)	19 (14-25)	22 (13-30)
**Years working in a FPC**	9 (2-12)	9 (2-15)	7 (2-11)	8 (2-12)
**FPCs Location**				
Paris	15 (23%)	6 (21%)	8 (36%)	1 (7%)
Seine-Saint-Denis	49 (77%)	22 (79%)	14 (64%)	13 (93%)
Centers characteristics*				
Hospital-based center	17 (27%)	7 (25%)	7 (32%)	3 (21%)
Community Health center	19 (30%)	12 (43%)	2 (9%)	5 (36%)
Maternal and child protection center	25 (39%)	9 (32%)	10 (46%)	6 (43%)
Other	7 (11%)	4 (14%)	3 (14%)	0
**Employment status in FPC**				
Full time	26 (41%)	8 (29%)	8 (36%)	10 (71%)
Part time	38 (59%)	20 (71%)	14 (64%)	4 (29%)
**Women seen per week in FPC**	25 (15-40)	30 (20-43)	23 (15-40)	17 (10-24)
**Ever heard about PrEP**	64 (100%)	28 (100%)	22 (100%)	14 (100)
**Ever prescribed PrEP**	10 (16%)	10 (36%)	0	0
**Activities in your FPC….**				
Offering PrEP services	18 (28%)	10 (36%)	4 (18%)	4 (29%)
Referring women needed PrEP	48 (75%)	22 (79%)	19 (86%)	7 (50%)
Offering informational material about PrEP	28 (44%)	15 (54%)	10 (46%)	3 (21%)
Collaboration with communities	22 (34%)	11 (39%)	5 (23%)	6 (43%)
**In our practice at FPC….**				
Ever initiated a PrEP conversation with a woman	27 (42%)	14 (50%)	8 (36%)	5 (36%)
Ever been asked about PrEP by a woman	16 (25%)	9 (32%)	4 (18%)	3 (21%)
Ever given a woman an appointment for PrEP	18 (28%)	9 (32%)	7 (32%)	2 (14%)
Ever referred a woman to another center to take PrEP	25 (39%)	10 (36%)	10 (46%)	5 (36%)
Ever prescribed PrEP to a woman	6 (9%)	6 (21%)	0	0

*FPC*, family planning center; *PrEP*, pre-exposure prophylaxis.

*Providers can work part-time in different FPCs.

### Experience with PrEP delivery in FPCs

Although all providers were aware of PrEP, 72% reported that their FPCs did not offer PrEP services, and three-quarters referred women to other centers when PrEP was needed. Forty-four percent of providers indicated that their center offered informational materials about PrEP. In their clinical practice, 58% of providers never discussed PrEP with a woman; 25% had been asked about PrEP by a woman; and about one-third had either referred women to other centers for PrEP or scheduled a PrEP appointment to initiate it. Among the 26 physicians, 6 (23%) had previously prescribed PrEP to a woman (Table 1), but only two had done so in the past 6 months.

### Providers’ perceptions of PrEP

Of the 64 providers, nearly all (98%) considered HIV prevention education to be somewhat or highly essential during FPC visits, with 94% believing that PrEP education is a crucial component of this. Regarding oral PrEP’s effectiveness, 92% of providers thought it was highly effective in preventing HIV acquisition among women who take it daily as prescribed. Nurses were less likely than physicians or midwives to perceive PrEP as highly effective (67% vs. 100% vs. 71% respectively, Fisher’s exact test *p* = 0.01). Over two-thirds (67%) of providers thought it was very unlikely or unlikely that women would increase sexual risk behaviors while on PrEP. A majority (64%) of providers reported feeling somewhat or very comfortable discussing PrEP with women.

### Providers’ knowledge about PrEP

**Fig 1:**
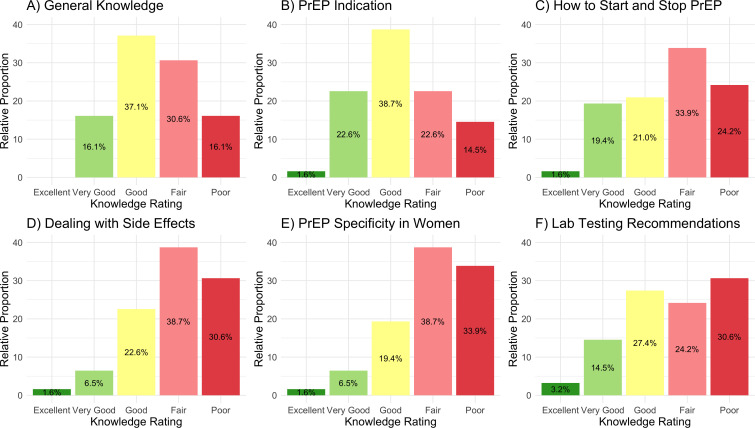
Providers’ knowledge about PrEP.

Forty-two percent of providers reported that they had already received PrEP training. Fig 1 illustrates the level of PrEP knowledge across several dimensions. Overall, 16% of providers reported having poor general knowledge about PrEP, and about one-third reported poor knowledge in specific domains such as managing side effects, laboratory test recommendations, or PrEP guidelines specific to women. Nurses were more likely than physicians or midwives to report poor general knowledge about PrEP, though this was not statistically significant (Nurse: 56%, Midwives: 10%, Physician: 12%, Fisher’s exact test *p* = 0.07).

### Barriers to PrEP use

**Fig 2:**
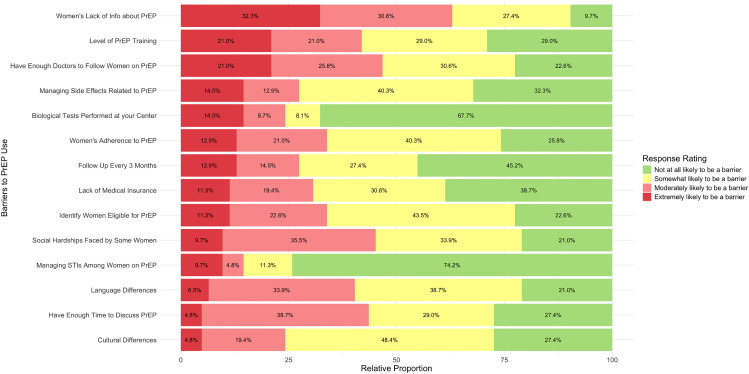
Providers’ perception of different barriers to PrEP use in women.

Fig 2 displays the perceived barriers to PrEP delivery in FPCs, ranked from most to least likely. The proportion of professionals who perceived the proposed items as extremely likely to be a barrier varied from 5% to 32% for each item. The top five items identified as extremely likely to be barriers were women’s lack of information about PrEP (32%), the low level of PrEP training among providers (21%), not having enough physicians to prescribe PrEP (21%), managing side effects related to PrEP (15%), and the need to carry out laboratory tests on site (15%). Perceptions of barriers differed among healthcare professionals, resulting in different rankings of the top three barriers.

### Facilitators to PrEP use

**Fig 3:**
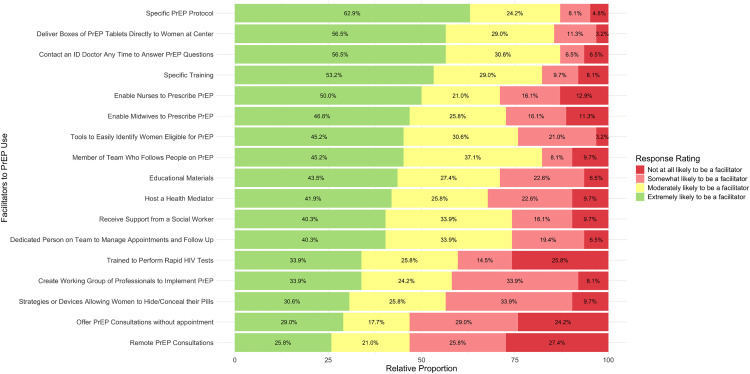
Providers’ perception of different facilitators to PrEP use in women.

Fig 3 displays the perceived facilitators to PrEP delivery in FPCs, ranked from most to least likely. The proportion of providers who perceived the proposed items as extremely likely to be facilitators varied from 26% to 63% for each item. The top five items identified as extremely likely to be facilitators were having specific PrEP protocols (63%), delivering PrEP boxes directly in the FPCs to women (57%), being able to contact an infectious disease physician at any time to answer PrEP questions (57%), having a specific PrEP training (53%), and enabling nurses and midwives to prescribe PrEP (50%).

## Discussion

In 2016, France became the first European country to fully subsidize PrEP for HIV prevention [29]. Yet, nearly a decade later, our findings reveal that PrEP remains underprovided in FPCs in the city of Paris and SSD County, two areas with a high HIV incidence and a large population of WMSSA [30]. Only one-third of surveyed providers reported offering PrEP within their centers, despite widespread recognition of its alignment with the mission of FPCs. This disconnect between principle and practice highlights the importance of examining the factors shaping PrEP implementation in these settings, including provider knowledge, perceptions, and the barriers and facilitators influencing delivery.

Providers overwhelmingly view PrEP as a valuable tool for HIV prevention. However, this positive perception contrasts with limited knowledge and training in our survey, highlighting a critical barrier at the provider level. The lack of adequate training, education, and standardized protocols, particularly regarding PrEP use for women (e.g., during pregnancy or breastfeeding), undermine providers’ confidence in discussing and prescribing PrEP, a challenge consistently documented in prior studies [31–33]. While physicians tend to be more informed, midwives and nurses, who predominantly staff FPCs, have received far fewer training opportunities. This disparity is particularly concerning given the introduction of new PrEP modalities like long-acting injectables, which are increasingly favored by women in France [34]. Some centers have attempted to address these gaps by referring women to external PrEP-providing sites, but this approach has been associated with significant drop-off along the PrEP care continuum, when not supported by a dedicated PrEP navigation system [35,36]. Conversely, offering same-day PrEP services within FPCs has been shown to enhance women’s engagement and access, aligning with their preference to receive PrEP where they already access care [37,38]. Addressing these training deficiencies through tailored programs for all providers in FPCs is essential for successful PrEP scale-up.

Providers’ hesitation to prescribe PrEP is further reinforced by low demand among women attending FPCs, creating a self-perpetuating cycle of underuse in these settings. Limited awareness of PrEP among women emerged as a top barrier identified by FPC providers. Although studies show that women express strong interest in PrEP once informed [34,39], their initial lack of awareness highlights the urgent need for targeted educational campaigns and outreach programs to bridge this gap. Community outreach, social media initiatives, and partnerships with women’s health organizations could effectively generate demand and improve uptake among FPC attendees.

Beyond individual knowledge and perceptions, operational and systemic barriers further complicate PrEP delivery. Providers cited a shortage of doctors available to prescribe PrEP for women, along with concerns about increased workload and resource constraints, challenges that align with findings from PrEP implementation studies in sexual and reproductive health centers from Africa and the U.S. [14,17,18,40–42]. Increasing PrEP uptake in FPCs may require dedicated human resources and the adoption of task-shifting strategies, such as empowering midwives and nurses to initiate or refill PrEP prescriptions. These approaches, along with client-centered interventions like HIV self-testing and mobile health tools, have been shown to optimize provider workflow and improve PrEP delivery in both high- and limited-resource settings globally [43–46]. The heterogeneity of FPCs in terms of on-site laboratory and pharmacy services also adds another layer of complexity. While HIV rapid testing is universally available in FPCs, not all recommended follow-up tests, such as STI screening or creatinine monitoring, can be conducted on-site. This limitation has driven the development of point-of-care testing solutions, such as for creatinine, to facilitate PrEP implementation in resource-limited environments [47]. Centers lacking laboratory or pharmacy infrastructure will need to explore alternative strategies, such as adopting point-of-care STI testing, establishing centralized laboratory partnerships, stocking PrEP medications for direct dispensing, or collaborating with local pharmacies.

The limited delivery of PrEP in FPCs underscores how the cumulative impact of these multi-level barriers has prevented providers’ commitment from being translated into actionable, women-centered PrEP services. Nevertheless, providers’ recognition of PrEP’s relevance, along with the broad support for proposed interventions observed in both this survey and the qualitative analyses, provides a strong foundation for future implementation efforts [48]. Providers endorsed a wide range of strategies to facilitate PrEP integration in their centers such as comprehensive training and mentorship, educational materials to raise PrEP awareness among women, tools to identify women with PrEP needs, and policy changes to expand the range of PrEP prescribers. These proposals align with successful strategies from women-focused PrEP initiatives in Africa and the U.S., where multifaceted interventions have demonstrated promise in increasing both uptake and adherence [14,15,49,50]*.* This information can be leveraged to inform the design of implementation strategies to effectively address and fill the PrEP delivery gap among women.

## Limitations and future directions

Our study has several limitations. First, not all the providers approached through the mailing list responded to the survey. Of those contacted, only 35% opened the questionnaire and 23% completed it in full. Providers who are more engaged or interested in PrEP might have been more likely to respond, which may have introduced selection bias. Second, our study focused on FPC providers working in two HIV transmission hotspots of the Paris region. Providers’ practices and the characteristics of women attending these centers may not be aligned with those of other FPCs, which could limit the generalizability of our results. Third, while the use of self-administered questionnaires may encourage more honest responses by reducing interviewer bias and increasing anonymity, the data collected were self-reported and thus remained subject to potential recall and social desirability biases, particularly for questions not using a Likert scale format. Providers might overestimate their knowledge or underreport their discomfort with providing PrEP. Fourth, providers from the same center could participate in the study, which may have led to an overrepresentation of barriers and facilitators perceived in larger centers compared to smaller ones or other inequalities in FPC representation. This may have introduced bias in our findings. Lastly, the limited sample size only allowed for a descriptive analysis, preventing exploration of individual or center-related factors associated with the perceived barriers or facilitators.

Despite these limitations, we believe the study provides valuable insights into healthcare providers’ experiences and attitudes toward delivering PrEP within French FPCs. These perceptions carry important implications for the future of PrEP implementation for women in the Paris region. To bridge the PrEP delivery gap in French FPCs, future efforts should prioritize multi-level, adaptive implementation strategies grounded in the barriers and facilitators identified in these centers. Key actions include implementing tailored training programs for all providers, launching women-focused demand-generation campaigns with support from community organizations, and adopting task-shifting models to enable midwives and nurses to prescribe and manage PrEP. These strategies will help integrate PrEP into routine sexual and reproductive health services, fostering equitable access to PrEP and resulting in a significant impact on women’s health.

## Conclusions

Providers in FPCs within Paris and Seine-Saint-Denis County demonstrate variable levels of knowledge, comfort, and experience in delivering PrEP to women*,* which may have contributed to its inconsistent delivery in these settings. Despite recognizing the value of PrEP and its alignment with the FPC mission, providers reported several barriers to implementation, including issues at the provider and client levels, center-level challenges, and systemic obstacles. While various barriers to PrEP delivery were identified, providers endorsed a wide range of interventions to increase PrEP delivery in their centers. These data can inform the design of tailored and multi-level implementation strategies to bridge the PrEP gap in FPCs.

## Supporting information

S1 Survey Provider survey. Full survey in English.
(DOCX)

S2 File Inclusivity in global research checklist. Completed checklist for ethical, cultural, and scientific considerations specific to inclusivity in global research.
(DOCX)
