# Supplementary material for: A cross-sectional survey to explore healthcare providers’ experiences and attitudes toward HIV pre-exposure prophylaxis for women in family planning centers of Greater Paris
Source: PLoS One. 2026 Jan 16;21(1):e0337510. doi: 10.1371/journal.pone.0337510 (PMC12810802; doi:10.1371/journal.pone.0337510)
Supplement: S1 Survey — Full survey in English. (DOCX) [file pone.0337510.s001.docx]

*PrEParez-vous!*

Providers Questionnaire

Version 1.0 - September 19^th^, 2023

# Study eligibility

To be sure that you can participate in the study, we would like to ask you a few questions.

1. How old are you today? [Text] (Age < 18 years = non-eligible)
2. What is your job?
   Doctor

Midwife
Nurse

Family therapist (=non-eligible)

Psychologist (=non-eligible)
Social worker (=non-eligible)

Others (=non-eligible)

1. Do you work in a center of the family planning (CPEF) of Paris and Seine-Saint-Denis? Yes / No (No = non eligible).

Non-eligible: Thank you for your time. Unfortunately, you are not eligible for this study.

Eligible: You are eligible to complete the survey. We are very interested in your opinions and experience. There are no wrong answers. If you do not want to answer a specific question, just skip or choose "Don't know" option when present and continue. We expect this survey to take no more than 20 minutes. Thank you again for your time and input.

# Participants’ characteristics and activities:

1. What is your gender?
   Male

Female

Other

1. How many years of practice do you have?

0 – 5 years

6 – 10 years

11 – 20 years

> 20 years

1. In which kind of centers do you work? [Only one choice]

CPEF in an hospital

CPEF in a municipal Health Center – *Centre municipal de santé*

CPEF

Sexual Health Clinics – *Centre de santé sexuelle*

Mother and child protection center – *Protection maternelle et infantile*

1. Where is located your center?
   Paris 🡪 If Paris, neighborhood - *Arrondissement*: [Open text]

Seine-Saint-Denis

1. What is your employment status?

Full-time

Part-time

1. How long have you worked in the CPEF?

0 – 5 years

6 – 10 years

11 – 20 years

> 20 years

1. How many women in CPEF do you see per week?

1-4

5-9

10-19

> 20

1. Currently, is the CPEF where you work offer PrEP services? Yes/No/I don’t know
2. Do you work with a center where you can refer women who need PrEP? Yes/No/I don’t know
3. Do you provide specific informational materials (brochure, video….) about PrEP in your center? Yes/No/I don’t know
4. Do you work with local communities or organizations in your CPEF? Yes/No/I don’t know

# PrEP Awareness:

1. Before today, have you heard about HIV Pre-exposure prophylaxis? Yes / No (if no : go to question 30)
2. Have you ever had specific training about PrEP? Yes / No
3. Have you ever initiated a conversation about PrEP with a woman in the CPEF where you work? Yes / No
4. Have you ever been asked about PrEP by a woman in the CPEF where you work? Yes / No
5. Have you ever advised a woman to take PrEP in the CPEF where you work? Yes / No
6. Have you ever referred a woman to another center to take PrEP? Yes/No
7. Have you ever prescribed PrEP? Yes/No
8. Have you ever prescribed PrEP to a woman in the CPEF where you work? Yes/No (If Yes : go to question 23, No : go to question 24).
9. How many prescriptions of PrEP in CPEF have you done for women in the past six months?

# PrEP knowledge:

How would you rate your knowledge of PrEP in the following areas:

|  | **Excellent**  Expert-level, up-to-date in research | **Very good**  Proficient, not up-to-date in research | **Good**  May not be as up-to-date and would likely review prior to prescribing | **Fair**  Review prior to prescribing | **Poor**  Heard of it |
| --- | --- | --- | --- | --- | --- |
| 1. General knwoledge |  |  |  |  |  |
| 1. PrEP Indication |  |  |  |  |  |
| 1. How to start and stop PrEP |  |  |  |  |  |
| 1. Dealing with side effects |  |  |  |  |  |
| 1. PrEP specificity in women (dealing with contraception,   pregnancy…) |  |  |  |  |  |
| 1. Lab testing recommended |  |  |  |  |  |

# Providers’ attitudes regarding HIV PrEP:

1. Do you think HIV prevention education is an essential part of a family planning (FP) visit?

Not at all essential

Somewhat not essential

Somewhat essential

Highly essential

1. Do you think PrEP education is an essential part of HIV prevention education at family planning (FP) visits?

Not at all essential

Somewhat not essential

Somewhat essential

Highly essential

1. How effective do you think PrEP is in preventing the acquisition of HIV among women who take it every day as prescribed?

Not effective

Somewhat effective

Moderate effective

Highly effective

1. How likely do you think a woman would be to increase her sexual risk-taking practices (eg, decrease condom use) as a result of being on PrEP?

Very unlikely

Unlikely

Somewhat likely

Likely

1. If you identified a patient at high risk for HIV acquisition, how would you rate your level of comfort discussing PrEP?

Very uncomfortable

Somewhat uncomfortable

Somewhat comfortable

Very comfortable

1. If you identified a patient at high risk for HIV acquisition, how would you rate your level of comfort prescribing PrEP?

Very uncomfortable

Somewhat uncomfortable

Somewhat comfortable

Very comfortable

# Barriers to PrEP use:

Could you rate the extent to which each of the following statements represents a potential barrier to implementing or strengthening PrEP prescription in your CPEF:

1 – Not at all likely to be a barrier (*Je pense que ce n’est pas un obstacle à la mise en œuvre de la PrEP dans mon centre.)*

2 – Somewhat likely to be a barrier

3 – Moderate likely to be a barrier

4 – Extremely likely to be a barrier 4 (*Je pense que c’est un obstacle majeur à la mise en œuvre de la PrEP dans mon centre.)*

1. Lack of provider training and education regarding PrEP.
2. Lack of clinical guidelines and protocol in my center for prescribing and monitoring PrEP.
3. Limited availability of PrEP-related resources for women.
4. Additional time required to discuss and provide PrEP with women.
5. Absence of awareness and willingness of women to take PrEP.
6. Discussing sexuality and HIV risk factors with women.
7. Identifying women who need PrEP.
8. The number of people who can prescribe PrEP in my center.
9. Not having a place to refer women who need PrEP.
10. Seeing women every 3 months for the PrEP follow-up.
11. Obtaining HIV tests and STI screening every 3 months.
12. Not being able to perform biological tests for PrEP on site.
13. Not being able to directly deliver PrEP bottles to women.
14. Not having a pharmacy on-site to facilitate PrEP delivery.
15. Managing STIs among women taking PrEP.
16. Dealing with the potential side effects related to PrEP.
17. Providing PrEP for women facing social hardships.
18. Providing PrEP for women with no insurance on the precarity budget.
19. Providing PrEP on the same day of the visit in the CPEF.
20. Prioritizing PrEP over other medical or social issues.
21. Communication with women not speaking French.
22. Cultural misunderstanding with some women.
23. Please share any other factors that could act as a barrier to PrEP prescription in your center. [Open Text]

# Facilitators to PrEP use:

Could you rate the extent to which each of the following statements could potentially act as a facilitator to implementing or strengthening PrEP prescription in your CPEF:

Facilitators

1 – Not at all likely to be a facilitator *(Je pense que cette mesure ne va pas aider à la mise en œuvre ou au renforcement de l’activité de PrEP dans mon centre).*

2 – Somewhat likely to be a facilitator

3 – Moderate likely to be a facilitator

4 – Extremely likely to be a facilitator – (*Je pense que cette mesure peut beaucoup aider à la mise en œuvre ou au renforcement de l’activité de PrEP dans mon centre).*

1. Receive specific training and education about PrEP.
2. Have a specific protocol for PrEP in my center.
3. Offer educational materials (video, brochure) to women in my center.
4. Have someone in my team that has already prescribed or has experience with PrEP.
5. Use specific tools (short questionnaire, medical report….) to detect women needing PrEP.
6. Be assisted by a PrEP coordinator in managing appointments, monitoring women, and providing treatments.
7. Have a dedicated PrEP center where I can easily refer women who need PrEP.
8. Have a hot line with ID physicians to answer PrEP prescribing and management questions.
9. Delivering PrEP bottles directly in my center.
10. Use telemedicine or virtual care platforms to make PrEP visits.
11. Work with peer support groups and associations to help women in their HIV prevention efforts.
12. Staff meetings or case discussions to share experiences and best practices related to PrEP provision.
13. Offer flexible clinic hours or walk-in appointments for PrEP visits.
14. Receiving support from a social assistant or a social worker who can manage social hardships in women.
15. Strategies or devices allowing women to hide/ concealed their pills.
16. Enable midwifes to prescribe PrEP in CPEF.
17. Enable nurses to prescribe PrEP in CPEF.
18. Please share any other factors that could facilitate PrEP prescription in your center.

[Open Text]

The questionnaire is now complete. As part of this study, we also offer healthcare professionals who wish to participate in a discussion group led by infectious diseases physicians. These discussion groups will be made up of ten healthcare professionals working in the CPEF/CSS of Paris and Seine-Saint-Denis, and will last one hour. The discussion will aim to better understand your perception of the implementation of PrEP in your center, to collect the difficulties you face in promoting this device and to discuss potential solutions to facilitate the use of PrEP in the CPEF / Sexual Health Centers of Paris and Seine Saint Denis. You will find full details of these discussion groups in the information leaflet you received. Compensation of 75 euros is provided for healthcare professionals taking part in the discussion groups outside their working hours.

77. Are you interested in taking part in the focus group?

- No -> We would like to thank you for your participation in this study. The results of this survey will be shared with you via the management of the CPEF / Centre de santé sexuelle de Paris et de Seine-Saint-Denis.

- Yes -> Please send an e-mail to Victoria, the study investigator, at victoria.manda@aphp.fr, stating your full name, profession, telephone number and place of practice. We would like to thank you for your participation.
